# Interview strategies for gender-diverse patients: Moving beyond “Men, women, or both?”

**DOI:** 10.1097/01.NPR.0000000000000443

**Published:** 2026-05-26

**Authors:** Roslyn M. Seitz, Miles Stetz Harris, Angela Jarman

**Affiliations:** **Roslyn M. Seitz** is a Health Sciences Assistant Clinical Professor in the Betty Irene Moore School of Nursing at the University of California, Davis in Sacramento, CA.; **Miles Stetz Harris** is a Health Sciences Assistant Clinical Professor in the Betty Irene Moore School of Nursing at the University of California, Davis in Sacramento, CA.; **Angela Jarman** is an Associate Professor in Emergency Medicine at the University of California, Davis.

**Keywords:** gender diverse, gender identity, nonbinary, sexual health history, transgender, trauma-informed care

## Abstract

This article supports nurse practitioners in creating a gender-inclusive clinical practice by offering up-to-date and trauma-informed interview techniques. Historical context of the care of gender-diverse patients is included to help the reader better understand the impact of past trauma and associated health disparities.

The common interview question, “Do you have sex with men, women, or both?” is insufficient for accurate clinical risk assessment. This phrasing assumes partners are cisgender (ie, not transgender or gender diverse) and encourages clinicians to infer sexual behaviors based on partner gender, rather than directly assessing the behaviors and anatomy relevant to pregnancy and infection risk. Although this question was originally recommended in the 2006 sexually transmitted infection (STI) guidelines,^1^ its continued use can contribute to incomplete histories and inaccurate risk assessment. Accurate assessment requires distinguishing a patient's gender identity, the gender(s) of their partners, the sexual practices they engage in, and the anatomy involved in those practices.

High-quality health histories are vital to an accurate diagnosis and treatment plan, yet many providers lack education and experience in caring for transgender and gender-diverse (TGD) patients, resulting in negative health care experiences.^2^-^4^

Applying trauma-informed interview strategies can improve patient experiences and patient-centered health outcomes. This article provides up-to-date strategies to collect an accurate and inclusive sexual health history for patients of all genders.

## ESTABLISHING RAPPORT

### Use the patient's name and pronouns

Using the name a patient uses (which may differ from the name in the medical record) and their pronouns is a key strategy that clinicians can use to convey respect, establish rapport, and demonstrate competency in gender-inclusive care.^2^-^5^ Asking directly and using this language consistently throughout the encounter supports a therapeutic alliance. Wearing a pronoun pin and sharing your own pronouns helps normalize this practice.

Suggested language:
*“What name would you like me to use?”*
*“What are your pronouns?”*

### Communicate using current terminology

Using patient-centered terminology helps build a strong therapeutic alliance. Communication should reflect how TGD people discuss their bodies and sexual practices. Clinicians should use the language a patient uses for themselves and remain familiar with commonly used and evolving terminology.

### How to ask sensitive questions

Six essential principles of trauma-informed practice are safety; choice; collaboration; trustworthiness; empowerment and choice; and consideration of cultural, historical, and gender factors. Application of trauma-informed strategies can reduce or avoid traumatization and retraumatization of the patient.^3^ The following approach provides a practical way to apply these principles during the clinical interview.

1. EVALUATE which components of the health history are needed.

Some presentations may have a clear connection to sensitive questions, whereas others do not. TGD patients may have previously been asked unnecessary or intrusive questions about their bodies and sex lives.^4^ These experiences can be traumatic and decrease willingness to seek future care.^2^

2. EXPLAIN to the patient why you need to ask questions.

Especially when the relationship between the patient's concern and the sensitive question is less obvious, it is essential to appropriately frame questions and educate the patient about the purpose of the question. Gender-related medical misattribution and invasive questioning (GRMMIQ), in which an individual is asked invasive and unnecessary questions and/or has their symptoms misattributed to their gender identity or transition, is a form of discrimination often experienced by TGD patients.^4^ Appropriately framing personal questions can help the patient directly correlate the query for information with their medical care and can avoid the appearance of impropriety.

3. EMPOWER the patient by asking permission.

Requesting permission to discuss a subject applies trauma-informed techniques and emphasizes patient autonomy.^5^ Patients may decline to answer a question. If this occurs, it is important for the clinician to explain (or reexplain) the reasoning for history collection and its role in their workup.

### Reestablishing rapport

If you make a mistake, such as using an inappropriate pronoun, name, or term, it is essential to apologize and keep the conversation focused on the patient. Apologies should be sincere and brief.

Suggested language: *“I am sorry for using the wrong pronoun/name/term. I did not mean to be disrespectful.”*

## FIVE PS OF THE SEXUAL HEALTH HISTORY

The Five Ps framework can be adapted for taking sexual health histories of TGD patients.^6^ In this context, understanding a patient's current anatomy (sometimes referred to as an “organ inventory”) is an important component of accurately assessing risk and guiding care. Other frameworks, like “Taking Routine Histories of Sexual Health: A System-Wide Approach for Heath Centers,” may also be tailored for patient-centered care.^7^

### Partner(s)

Asking about sexual partners helps guide assessment of STI and pregnancy risk, as well as broader sexual health and care needs. The term “partners” is recommended as gender-neutral language.

Suggested language:
*“Can you tell me about your sexual partners, including how they describe their gender (for example, cisgender, transgender, or nonbinary)?”*
*“Is there anything about your partners' bodies or anatomy that would be helpful for me to know for your care?”*
*“Do your sexual partners have other sexual partners?”*

Patients who have been assaulted, coerced, molested, trafficked, and/or raped may not include the assailants as partners. Additionally, those engaged in sex work may not include clients as partners. Clinicians should consider asking about these experiences in a trauma-informed manner, as they are common and may impact risk assessment.

### Practices

Sexual practices should not be inferred from a patient's sexual orientation and should instead be assessed directly. The question “are you sexually active?” is often vague in terms of activities and timeframe; it is more useful to ask about specific sexual practices within a defined period. Patients who identify as gay or lesbian may still engage in sexual activity that could result in pregnancy, underscoring the importance of direct assessment. Sexual practices may also be influenced by prior gender-affirming interventions; understanding a patient's current anatomy is important for risk assessment.

Suggested language:
*“When you have sex, do you tend to be insertive (top), receptive (bottom), or both?”*
*“What types of sex do you have (for example, vaginal, oral, or anal)?”*

### Protection from STIs

A conversation about protection from STIs, including HIV, is important, both to understand current risk as well as to prevent further infection through risk reduction, postexposure prophylaxis (PEP), and preexposure prophylaxis (PrEP). Previous studies have found that TGD patients experience higher rates of STIs, including HIV, in part due to structural barriers and disparities in access to care, and may benefit from site-specific (eg, pharyngeal and rectal) screening based on reported sexual practices.^8^-^10^ For more information about how to treat STIs and prevent HIV with use of PEP and PrEP, providers should refer to the CDC's guidelines and other relevant resources.

Suggested language:
*“Do you use condoms or other barrier protection when having vaginal, anal, or oral sex? How often?”*
*“Do you and your partner(s) use any other protection against STIs? If not, can you tell me more about that? If yes, what kind of protection do you use, and how often?”*

### Past history of STIs

Obtaining information about history of STIs, including HIV, can also be important in evaluating the etiology of a patient's signs and symptoms and interpreting laboratory results. Frame these questions so they are respectful and free from stigma.

Suggested language:
*“Have you been treated for any sexually transmitted infections in the past?”*
*“Have any of your previous partners told you they had HIV or another sexually transmitted infection, or that they had symptoms of a sexually transmitted infection?”*

### Pregnancy potential and intention

Pregnancy can be a sensitive subject for any patient and can be a source of past trauma. Patients may or may not desire pregnancy, so it is important to use neutral language when discussing the possibility of pregnancy. Clinicians should avoid asking, “Are you having any kind of sex that could get you pregnant?” because it requires patients to assess their own fertility.

TGD patients may be misinformed about fertility while using gender-affirming hormones.^11^ While gender-affirming hormone therapy may impact fertility, it does not reliably prevent pregnancy and should not be considered contraception.^11^,^12^ Additionally, clinicians should avoid assuming anatomical structures based on gender identity. As noted above, conducting an anatomical inventory (ie, organ inventory) can help assess pregnancy potential and inform diagnostic workup.

Suggested language:
*“I want to ensure that your treatment is appropriate for your body. Can I ask about anatomy (for example, uterus) that is relevant to your care?”*
*“Do you use any method to prevent pregnancy?”*
*“Do you have any partners whose bodies make sperm?”*

## KEY POINTS

- Establish good rapport. Use the patient's preferred terminology and pronouns.
- When you make a mistake, recognize the error, apologize, and focus on patient care.
- Remember to EVALUATE, EXPLAIN, and EMPOWER patients when asking sensitive questions.
- Avoid gender-related medical misattribution and unnecessary or invasive questioning.
- Use a trauma-informed approach in all aspects of patient care.
- Use the suggested language in this article or tailor to the patient's unique circumstances.
